# Group elicitations yield more consistent, yet more uncertain experts in understanding risks to ecosystem services in New Zealand bays

**DOI:** 10.1371/journal.pone.0182233

**Published:** 2017-08-02

**Authors:** Gerald G. Singh, Jim Sinner, Joanne Ellis, Milind Kandlikar, Benjamin S. Halpern, Terre Satterfield, Kai Chan

**Affiliations:** 1 NEREUS Program, Institute for the Oceans and Fisheries, The University of British Columbia, Vancouver, Canada; 2 Institute for Resources, Environment, and Sustainability, The University of British Columbia, Vancouver, Canada; 3 Cawthron Institute, Nelson, New Zealand; 4 King Abdullah University of Science and Technology (KAUST), Red Sea Research Centre, Thuwal, Saudi Arabia; 5 National Center for Ecological Analysis and Synthesis, Santa Barbara, California, United States of America; 6 Bren School of Environmental Science and Management, University of California, Santa Barbara, California, United States of America; 7 Imperial College London, London, United Kingdom; US Army Engineer Research and Development Center, UNITED STATES

## Abstract

The elicitation of expert judgment is an important tool for assessment of risks and impacts in environmental management contexts, and especially important as decision-makers face novel challenges where prior empirical research is lacking or insufficient. Evidence-driven elicitation approaches typically involve techniques to derive more accurate probability distributions under fairly specific contexts. Experts are, however, prone to overconfidence in their judgements. Group elicitations with diverse experts can reduce expert overconfidence by allowing cross-examination and reassessment of prior judgements, but groups are also prone to uncritical “groupthink” errors. When the problem context is underspecified the probability that experts commit groupthink errors may increase. This study addresses how structured workshops affect expert variability among and certainty within responses in a New Zealand case study. We find that experts’ risk estimates before and after a workshop differ, and that group elicitations provided greater consistency of estimates, yet also greater uncertainty among experts, when addressing prominent impacts to four different ecosystem services in coastal New Zealand. After group workshops, experts provided more consistent ranking of risks and more consistent best estimates of impact through increased clarity in terminology and dampening of extreme positions, yet probability distributions for impacts widened. The results from this case study suggest that group elicitations have favorable consequences for the quality and uncertainty of risk judgments within and across experts, making group elicitation techniques invaluable tools in contexts of limited data.

## Introduction

Policy and management concerns on issues as diverse as health, technology, and the environment require scientific input to understand the mechanisms, magnitudes and characteristics of emerging risks [1, 2]. A rapidly changing world has spurred research to assess the severity and uncertainty of effects of novel diseases and technologies, such as the risk posed by disease incursions and nanoparticles on human health, and climate change on ecosystems [3–5]. Emerging risks are typically characterized by sparse data on both likelihood and magnitude, necessitating a reliance on expert judgment to inform policy. Elicitation of expert judgement can yield useful and timely information in the face of novel, data-poor problems that require expeditious management decisions [2, 6]. Expert judgements, if elicited carefully, draw upon extensive experience, potentially providing policymakers with best available information on possible consequences, uncertainties, and tradeoffs of variables of interest [2, 3, 7, 8]. Cumulative anthropogenic impacts to the environment are one type of emerging and increasingly serious issue for policy, often requiring management when data is sparse. Policy makers therefore rely on expert elicitation processes to generate useful information.

In order to increase the quality of information derived from experts, researchers relying on expert judgement require knowledge about effective elicitation strategies [2, 7, 9]. Experts are, of course, human, and as such they are susceptible to cognitive biases and unreliable mental shortcuts [10–12]. Thankfully, research has established effective techniques to address these biases and generate more reliable estimates for management-relevant science questions. For example, to address overconfidence and other cognitive biases among experts, group elicitation procedures are often recommended. In groups, experts can challenge and corroborate each other’s ideas while clarifying vague terminology, and they are often forced to reevaluate the confidence placed in their individual elicited quantitative estimates [6, 13, 14]. Techniques designed to question confidence have been applied when experts have specific contexts for which to make estimates. For example, experts may be asked to provide quantitative estimates of extinction risk for particular species given specific conditions. However, experts may be required to specify the context of emerging risk before providing estimates, given the inherently high uncertainty in understanding emerging risks. For example, experts may be first asked to identify the prominent threats to a species, then quantify risk of each. In such cases, when experts are asked to parameterize *a priori* undetermined variables in an under-specified context, we do not know how techniques designed to question confidence then affect expert judgement.

This problem is thus: humans are imperfect intuitive statisticians, relying on cognitive heuristics to make judgements when confronting uncertainty [10, 15–17]. While heuristics can provide useful guidance given limited time [16], they can also lead to biased judgements based on the ease with which instances are recalled–the availability heuristic [18]–and provide quantitative estimates influenced by previous suggestions–the anchoring bias [17]. Experts are also often recognized as privileged holders of information and can display overprecision (a form of overconfidence) in their judgements, reporting confidence intervals for estimates that do not reflect the “true” answer [2, 19]. Group elicitation approaches are sometimes proposed to address these challenges, but have also been found to both attenuate bias and enhance understanding, or amplify bias and confound understanding, depending on the bias and the group process [20]. Group elicitation approaches (often modified from Delphi group approaches) can mitigate these biases, and specific approaches can even mitigate the influence of dominant voices drowning out less assertive ones [6, 9]. Group settings allow experts to challenge each other and are more likely to induce an individual expert to rationally reassess their judgements [13]. Diverse groups of experts are also more likely to challenge in-group thinking that can arise when experts from similar disciplines reinforce each other’s judgements [21, 22]. However, group decisions can easily lead to “groupthink” effects, where groups tend towards uniformity and censorship, particularly when groups are made of like-minded individuals that crowd out dissenting voices [22–24]. Groupthink effects are also aggravated by difficult tasks, which can lower each expert’s sense of self-efficacy for critical evaluation, leading to credulous consensus (22, 23). For groups to generate reliable estimates, therefore, the elicitation process needs to challenge the confidence any expert has in their initial judgment and include diverse participants to mitigate groupthink effects.

This group-discussion effect has been tested and understood under contexts where experts are asked to provide quantitative estimates under conditions where context is fully specified thereby enabling parameterization [7]. However, there are many cases in environmental management where expert input is needed to help define a question before it can be answered [25]. For example, when managers are interested in addressing cumulative impacts, which risks to prioritize for management is rarely clear *a priori* [26]. Experts are needed first to identify the primary causes of cumulative impacts, and second to quantify the risk associated with these impacts. When faced with such a two-part problem, the problem of group members affecting each other might be exacerbated by the difficulty of the task. How does group elicitation affect expert responses with two-phased problems? To our knowledge, this is the first study to address group elicitation with two-phased problems by comparing expert responses before and after group elicitation.

If and when group processes do lead to unreliable judgements because the elicitation context is difficult, for example, understanding highly uncertain problems such as cumulative impacts, elicitation processes using individuals may be more useful provided other biases can be adequately managed. In light of these dilemmas, we imagine four possible outcomes for how group processes will affect expert estimates in these two-phased problems. The first outcome is that experts will suffer from groupthink, and both individual and group estimates will be more certain after a group process because groupthink minimizes conflict and decreases critical evaluation in experts while increasing confidence [27]. With this outcome, expert estimates of impacts will occupy a narrower prediction interval after a group process compared to predictions before, and experts’ best point estimates will be more similar to each other. The second possible outcome is that group deliberations will significantly shake the confidence of experts, whose judgements will show increased variation in both individual and group responses. In this case, expert estimates of impact will occupy wider prediction intervals and best point estimates will be more variable among experts after a group process. The third possible outcome is that group processes will lead to group polarization, whereby opinions among experts become more extreme and confident as like-minded subgroups reinforce each other and strongly disagree with other subgroups [28]. In this outcome, expert estimates of impact will occupy narrower prediction intervals and best point estimates will be more divergent among experts after group processes. Finally, the fourth possible outcome is that group processes will clarify initial misunderstandings, difference in available knowledge, or variation in terminology and underlying conceptual models, leading to convergence in understanding among experts, while still challenging individual experts [13, 29, 30]. With this outcome, expert estimates of impact will occupy wider prediction intervals and variation in best point estimates of impact will diminish post group process.

This study seeks to address these questions in a case where experts are asked to specify the most important risks and then score the impact from them, in the context of eliciting judgments about cumulative impacts on Tasman and Golden Bays in New Zealand. Specifically, we address these questions through an expert elicitation study designed to understand prominent risks to ecosystem services in coastal New Zealand. In this case, to understand how group deliberation affected expert consistency/variation and subjective uncertainty (an expert’s uncertainty in estimating risk), we asked experts to provide individual responses before and after a workshop with a diverse group of experts.

## Methods

Our case study in Golden Bay and Tasman Bay (New Zealand) included four major methodological steps. First, we identified local experts and solicited their expertise about major risks to four ecosystem services via an online survey. Second, we interviewed all of these experts individually, wherein we asked additional questions about mechanisms and magnitude of impacts. Third, we held a Delphi-like group workshop involving most interviewed experts, which involved a group deliberation and then individual re-scoring of impacts. Our design was focused on training and guiding experts through the problem context and data collection exercises. The opportunity to enable expert learning about and training for tasks is a major advantage of expert elicitation [2, 9]. Fourth, we analyzed the magnitude and uncertainty of impacts within and across experts. This study was approved by the UBC Behavioural Research Ethics Board (#H14-00042). In the five subsections below, we describe the study site and these four major steps.

### Study site

Tasman and Golden Bays are situated at the northern end of the South Island of New Zealand. We focus on impacts to fisheries, shellfish aquaculture, marine recreation, and existence values of biodiversity because these are all important uses/benefits in the bays.

Tasman and Golden Bays have a history of human alteration. Trawl fisheries have historically contributed to the transformation of the benthic habitat from a structurally complex environment with thick mussel beds and oyster reefs to a flat silty bottom [31]. Removal of forest cover for agriculture, plantation forestry, and urbanisation in the latter half of the nineteenth century have also affected the bays [31].

### Online survey

We recruited experts through recommendation by a leading research organization in the area (the Cawthron Institute). Experts were not just scientists; they were people who worked and recreated in, studied, or advocated for the area, which was important given the site specificity of both the ecosystem services and the anthropogenic impacts that occur there [8, 9, 32]. Expert competence was gauged based on years worked and studied within the bays (most had over a decade of experience), and recognition among the expert community through snowball sampling [33]. In total 42 experts were contacted and 20 took part in the elicitation (47.6% recruitment rate). Choosing experts with area-specific knowledge is important, as the opinions of experts without such knowledge can misrepresent the specific characteristics of the two bays [7, 34]. Local experts are still prone to cognitive biases, but structured elicitation processes (such as cross-examination by facilitators and other experts) can effectively circumvent or ameliorate these biases [8]. The expert group included five fisheries experts, three aquaculture experts, four recreation experts, and eight biodiversity experts (emphasizing that we were interested in the existence value of biodiversity).

First, each expert was asked to complete an online survey consisting of one question, which asked them to provide a ranked list of up to five risks they regarded as threatening a given ecosystem service. They were given a list of risks to choose from, with the option of adding others not indicated (Table 1). The pre-defined list consisted of ten broad activities and stressors, adapted from global and regional cumulative impact assessments (Halpern et al. 2008, Halpern et al. 2009), emphasizing broad categories. Though this initial list encouraged experts to consider risks broadly, experts were free to add activities and stressors not on the initial list. Five experts identified a total of eight additional activities and stressors not on our initial list.

**Table 1:** The list of ten activities and stressors initially provided for experts to rank, with the additional eight suggested by experts.

List of Activities and Stressors	
Initial List	Agriculture
	Pollution
	Coastal Structures
	Commercial Shipping
	Invasive Species
	Aquaculture
	Recreational Fishing
	Commercial Fishing
	Climate Change
	Human Trampling
Additional List	Disease
	Ocean Acidification
	Sedimentation
	Social Licence
	Nutrient Input
	Forestry
	Land Clearing
	Poor Regional Planning

### Individual interviews

All 20 experts who took part in the online survey were then interviewed (step 2). Experts were first asked to confirm their ranking, then asked why they ranked the way they did. As the study focus was on impacts to ecosystem services–encompassing biophysical and socioeconomic domains—experts were reminded that humans can cause impact through biophysical as well as socioeconomic means. The interviewer asked each respondent to explain the processes by which risks cause impact, and what aspects of risk they emphasized in their ranking (such as the scale of impact, if the ecosystem service was particularly vulnerable to it, etc.). Experts were then asked to provide estimates of impact of their ranked risks to the bays under current levels of activity for the immediate future (0–5 years). Experts were asked to provide an interval of values (i.e. a minimum and a maximum) they believe includes the level of impact to their specific ecosystem service, between scores of 0–1. A score of 0 indicates that the ecosystem service is unimpacted, while a score of 1 indicates that the ecosystem service is rendered unavailable for human enjoyment. They were also asked to provide a best estimate of impact. After completing these steps, experts were asked to indicate how confident they were in their judgement (from 0–100%) that their estimated interval included the true value, and asked to make sure they were at least 50% confident. This step forces experts to reevaluate the interval they provided, and often forces them to expand their interval [19]. Then experts were introduced to probability distribution functions (PDFs), and asked to draw a function representing the impact from each of their ranked stressors, with the ends of the interval indicating the minimum and maximum, and the best estimate indicating the mode. We had a facilitator train and assist experts in drawing PDFs, and provide feedback, to ensure that expert responses correctly captured the experts’ risk estimates [7, 35].

### Group workshop

Finally, we convened an expert workshop adapted from Burgman [6], Fischhoff [25], Fish [21], and Speirs-Bridge [19] designed to reduce prominent biases in expert elicitation, including availability, anchoring and adjustment, dominance, and overconfidence (providing narrow confidence bands). Fifteen experts previously interviewed attended a workshop following the interviews (6 biodiversity experts, 3 aquaculture experts, 3 fisheries experts, and 3 marine recreation experts). All experts save one were previously interviewed; one fisheries expert was asked to fill in on behalf of another expert who could not make it to the workshop. This new expert was briefed on the activities and background. These experts’ data were not analyzed for this study because we did not have data for them either before or after the workshop.

The workshop had four sessions, each dedicated to one ecosystem service. Each session was based on a Delphi method approach with facilitators prompting experts to defend their views to each other about the most important risks. All ecosystem service experts were invited to partake in each discussion; this was designed to reduce disciplinary bias and expand the scope of the problem being considered [21]. Though we allowed face-to-face interactions in facilitated groups, we maintained anonymity of expert responses by presenting aggregated results from individual interviews and then asking experts to provide their reconsidered responses individually. Each session ended with experts individually filling out response forms without discussion, which were then handed to the facilitators. On these response forms, experts were asked to again rank up to five risks to the ecosystem services, provide probability distribution functions for the level of impact, and indicate their confidence in the intervals, ensuring they were at least 50% confident in their interval.

### Analysis

Only data from experts who provided both pre-workshop and post-workshop estimates (n = 14, including 3 aquaculture experts, 2 fisheries experts, 3 recreation experts, and 6 biodiversity experts) were analyzed, and only on those risks that they scored both before and after the workshop. Expert consistency was analyzed in two ways. First, importance of risks within each expert domain was modeled to predict how the experts as a group would rank risks to particular ecosystem services using the Insertion Sorting Rank (ISR) algorithm for analyzing ranking data [36, 37]. We recorded a measure of expert homogeneity associated with the ISR algorithm, scored from 0.5 to 1, where 1 indicates total certainty that the model captures expert ranking as a group and 0.5 indicating complete uncertainty in ranking among experts captured by the model. This is a predictive algorithm based on observed expert ranks and not a comparative test among rankings, and it is not subject to sample-size effects. We use this analysis to compare homogeneity of experts. Small differences in the homogeneity score (~0.03) can represent differences in expert homogeneity, and we use the standard deviation of the distance between the final estimate of the homogeneity score and the score at each step in the iterative algorithm as a measure of score variation [38].

To assess between-expert consistency (vs variability) in responses, we analyzed the best estimates (modes of the elicited expert distribution) of impact from all experts before and after the workshop, and compared the consistency in scores. To do this, for each specific type of risk for each ecosystem service, we calculated the absolute value of the difference of individual scores from the mean score to generate a measure of difference among experts. We then compared the difference scores post-workshop from the difference scores pre-workshop. According to the impact scale experts used to judge activities and stressors, differences from the mean on the order of 0.1 represent a view that is 10% different from the mean expert judgement. Because our samples before and after the workshop were not independent and were shared with experts, we conducted a mixed-effect paired t-test of these differences for estimates before the workshop and after [39]. The paired before-after risks nested within experts were treated as random effects, and the difference score treated as a fixed effect. Some risks were named differently before and after the workshop by experts, as deliberation in the workshop reduced linguistic uncertainty and resulted in convergence of terminology. Based on the reasoning given in the interviews and workshop (and/or confirmed with specific experts following the workshop), some risks were combined. For example, in initial interviews many experts scored impacts from agriculture because of its contribution to sedimentation, whereas after the workshop these experts combined these risks and scored impacts from sedimentation only. In these cases, we included comparisons of impact scores between agriculture and sedimentation. Sample sizes for these analyses are 14 comparisons for aquaculture, 5 comparisons for fisheries, 9 comparisons for marine recreation, and 19 comparisons for biodiversity.

Subjective uncertainty in individual expert judgement was analyzed by comparing the intervals of impact for a given risk on a given ecosystem service by a given expert. Similar to the analysis on best estimates, we only include results from the 14 experts shared between the interview and workshop stages. We compared the range of intervals before and after the workshop by subtracting the interval of each post-workshop estimate from the interval of the corresponding pre-workshop estimate and using a mixed-effect paired t-test. We treated the before-after pair nested within an expert as a random effect and interval range as a fixed effect. In contrast to the analysis of best estimates, which compared individual estimates against a group mean, we could include intervals for activities and stressors that were not considered by multiple experts, meaning that our samples sizes were larger than for the previous analyses of best estimates. Sample sizes for analyses on intervals are 14 comparisons for aquaculture, 7 comparisons for fisheries, 11 comparisons for marine recreation, and 20 comparisons for biodiversity. Mixed-effect paired t-tests were conducted using the nlme package in R [40] and the rank modeling was conducting using the R package rankcluster [38].

## Results

### Among expert consistency

#### Ordinal consistency

**Fig 1:**
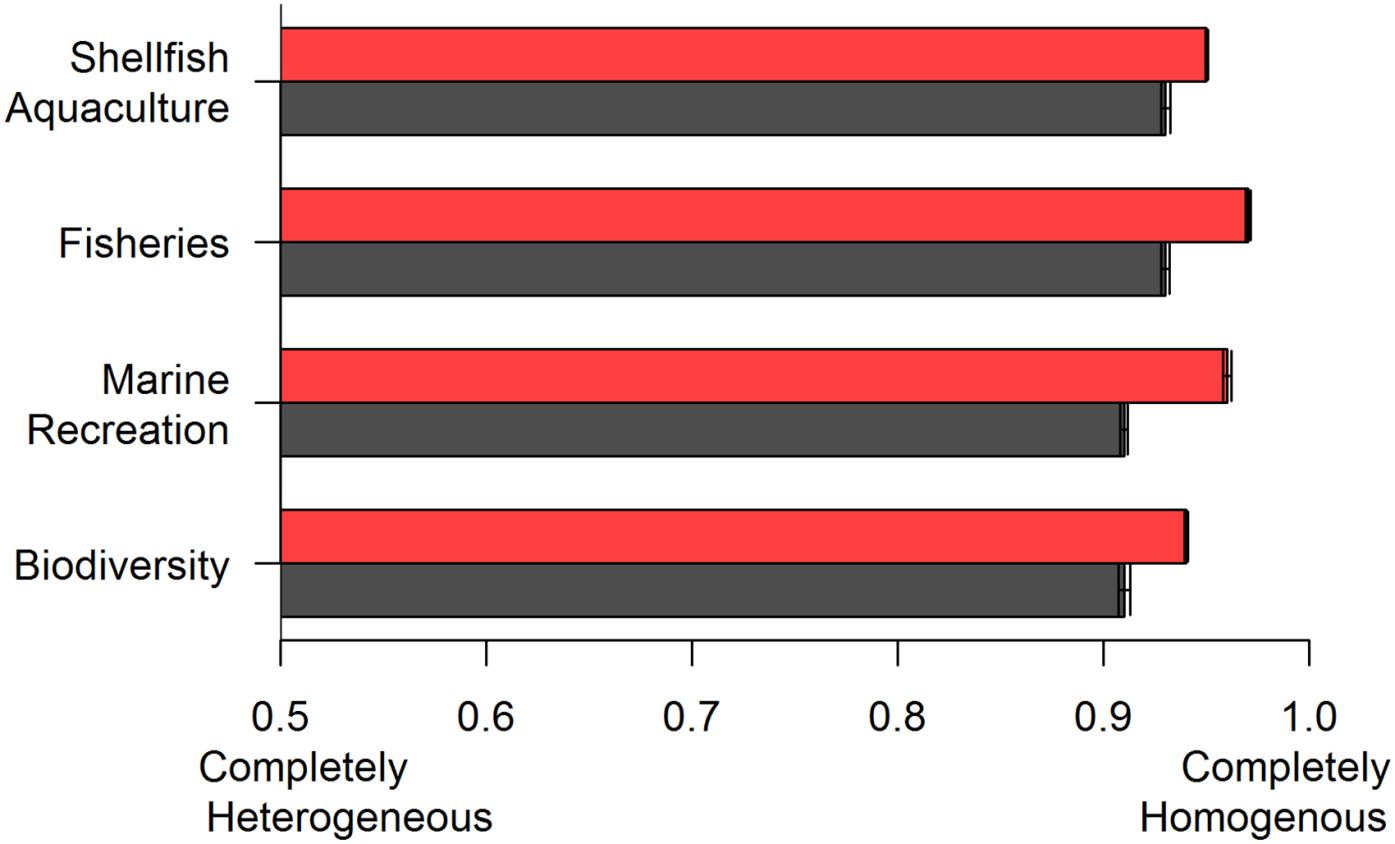
Homogeneity of expert rankings. Bars represent homogeneity scores before (grey) and after (red) the group workshop.

Following the expert workshop, experts displayed a more homogenous ranking of risks across all four ecosystem services compared to rankings by experts interviewed alone (Fig 1). The largest changes in consistency were found in marine recreation experts (homogeneity score change from 0.91 to 0.96) followed by fisheries experts (0.93 to 0.97), biodiversity experts (0.91 to 0.94), and finally aquaculture experts (0.93 to 0.95). Variation within these estimates was considerably smaller than between these estimates (ranging from 6.0×10^−4^–2.0×10^−3^).

After the workshop, experts were more likely to rank specific stressors highly, such as sedimentation and pollution, whereas before the workshop most experts pointed to activities that contribute to these stressors, providing a diverse list of activities such as agriculture, forestry, and land clearing. During interviews, one expert highlighted sedimentation as an important risk not included on the original list, indicating that many activities are primarily a risk through sedimentation. Indeed, many experts who raised concerns of agriculture, forestry, land clearing, and even dredge-based fisheries identified sedimentation as the primary reason they ranked these risks highly, with some suggesting runoff-based pollution and nutrient input are major considerations for the inclusion of land based activities. Through facilitated discussion, terminology was agreed and experts were able to clarify their meaning and focus their ranking on more specific stressors (Table 2). As a result, sedimentation moved up the rankings for all four domains, featuring as the top-ranked risk for both fisheries and biodiversity.

**Table 2:** Modeled rank of prominent risks to ecosystem services in Tasman and Golden Bays, New Zealand. The degree of expert homogeneity in ranking is provided under each ecosystem service and time (at interview stage or after workshop), and risks are ranked from greatest to lowest risk assessed.

	Aquaculture		Fisheries		Marine Recreation		Biodiversity	
	Interview	Workshop	Interview	Workshop	Interview	Workshop	Interview	Workshop
Homogeneity	0.93	0.95	0.93	0.97	0.91	0.96	0.91	0.94
1	Pollution	Invasive Species	Forestry	Sedimentation	Commercial Fishing	Pollution	Commercial Fishing	Sedimentation
2	Agriculture	Climate Change	Agriculture	Climate Change	Pollution	Human Trampling	Coastal Structures	Coastal Structures
3	Invasive Species	Pollution	Aquaculture	Aquaculture	Human Trampling	Shipping	Pollution	Pollution
4	Climate Change	Nutrient Input	Commercial Fishing	Commercial Fishing	Recreational Fishing	Sedimentation	Invasive Species	Invasive Species
5	Disease	Social License	Pollution	Pollution	Invasive Species	Climate Change	Climate Change	Commercial Fishing
6	Commercial Fishing	Sedimentation	Coastal Structures	Recreational Fishing	Aquaculture	Social License	Agriculture	Social License
7	Aquaculture	Aquaculture	Climate Change	Social License	Nutrient Input	Nutrient Input	Forestry	Human Trampling
8	Human Trampling	Disease	Invasive Species	Agriculture	Shipping	Commercial Fishing	Aquaculture	Climate Change
9	Ocean Acidification	Shipping	Recreational Fishing	Human Trampling	Climate Change	Recreational Fishing	Human Trampling	Forestry
10	Recreational Fishing	Recreational Fishing	Sedimentation	Forestry	Social License	Aquaculture	Recreational Fishing	Shipping
11	Sedimentation	Human Trampling	Social License	Shipping	Sedimentation	Invasive Species	Shipping	Nutrient Input
12	Social License	Commercial Fishing	Shipping	Invasive Species	Coastal Structures	Coastal Structures	Land Clearing	Poor Regional Planning
13	Shipping	Coastal Structures	Human Trampling	Nutrient Input			Sedimentation	Land Clearing
14	Coastal Structures	Ocean Acidification	Nutrient Input	Coastal Structures			Nutrient Input	Agriculture
15	Nutrient Input	Agricultures					Social License	Recreational Fishing
16							Poor Regional Planning	Aquaculture

In some cases, experts included entirely different risks in their rankings after deliberation. For example, in interviews marine recreation experts did not consider land-based drivers important (compared to their other top five activities and stressors), but when confronted with expertise in fisheries and biodiversity (who ranked sedimentation as the chief concern), recreation experts included sedimentation as one of the top 5 risks to marine recreation.

### Numerical consistency

Experts’ best-estimate impact scores were more consistent (with other experts) after the workshop than before (Table 3). This was the case for all four ecosystem services, and was statistically significant for each. The greatest difference in consistency between the interviews and workshop was for biodiversity (the mean±SE difference from the mean of pre-workshop scores was 0.076±0.027 greater than post workshop, p = 0.012), followed by marine recreation (0.072±0.024, p = 0.018), fisheries (0.06±0.02, p = 0.041), and finally aquaculture (0.044±0.014, p = 0.009). In a small number of cases, experts’ best estimates were further from the group average after the workshop than before. This occurred in two of fourteen comparisons for aquaculture experts, four of nineteen comparisons for biodiversity experts, and one of nine comparisons for marine recreation experts.

**Table 3:** Summaries of mixed-effect paired t-tests comparing the consistency in best estimate and interval for risks to the four ecosystem services before and after the workshop. The column “experts” refers to the number of experts in the analysis, while “comparisons” refers to the number of before-after comparisons included in the analysis (these were treated as nested random effects in the models). The column “difference” records the mean difference from the average best estimate (“best estimate” rows) as well as the mean interval length (“interval” rows) before compared to after the workshop. Positive “best estimate” differences indicate that the individual best estimates were more similar to the average after the workshop (than before); positive “interval” differences indicate that the interval range of estimates shrunk after the workshop. The column “df” reports the degrees of freedom in the analysis.

Ecosystem Service	Test	Experts	Comparisons	Difference	Standard Error	Df	t-value	P-value
**Aquaculture**	Best Estimate	3	14	0.044	0.014	13	3.095	0.009
	Interval	3	14	-0.091	0.042	13	-2.194	0.047
**Fisheries**	Best Estimate	2	5	0.060	0.020	4	2.979	0.041
	Interval	2	7	-0.121	0.049	6	-2.497	0.047
**Marine Recreation**	Best Estimate	3	9	0.072	0.024	8	2.978	0.018
	Interval	3	11	-0.118	0.051	10	-2.316	0.043
**Biodiversity**	Best Estimate	5	19	0.076	0.027	18	2.782	0.012
	Interval	6	20	-0.159	0.063	19	-2.524	0.021

### Within-expert subjective uncertainty

**Fig 2:**
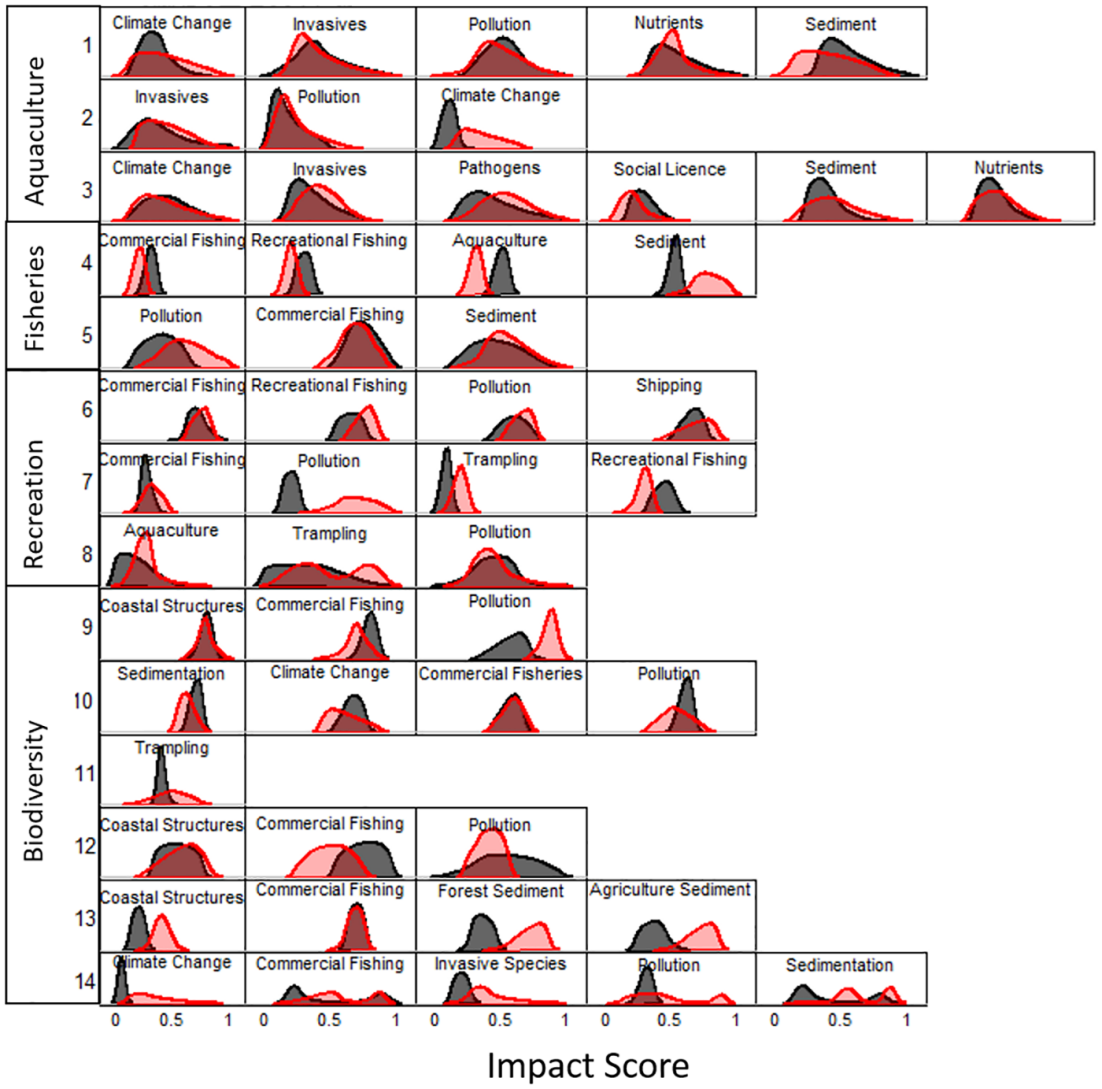
Probability distribution functions (PDFs) representing expert-derived estimates of impact from interviews (grey), and following the group workshop (red). Following the workshop, experts were more likely to provide wider intervals estimating impact from specific risks to ecosystem services compared to when interviewed prior to the workshop. Each row represents the paired estimates for a single expert (1–14). Experts are grouped by the ecosystem service of their expertise.

The mean size of interval that experts believe captures the true impact score of specific risks was larger post-workshop than in individual interviews, a statistically significant finding for all four ecosystem services (Table 3). The size of the interval (i.e. the difference between maximum and minimum estimates) increased the most for biodiversity experts (the mean±SE size of post-workshop intervals was 0.159±0.063 greater than pre-workshop intervals, p = 0.021), followed by fisheries experts (0.121±0.049, p = 0.047), marine recreation experts (0.118±0.051, p = 0.043), and finally aquaculture experts (0.091±0.042, p = 0.047). In a few cases, experts provided smaller intervals post-workshop than pre-workshop: three to fourteen assessments by aquaculture experts, two of twenty for biodiversity experts, and two of eleven for marine recreation experts (Fig 2).

## Discussion

The findings of our case study support the well-documented effect of group settings to reduce expert over-confidence and non-overlapping estimates among experts [2, 6, 19]. Moreover, our findings confirm that group elicitations can clarify misunderstandings and linguistic uncertainty, generating convergence in understanding of the problem [19, 29, 30]–the fourth (and most favourable) of the possible outcomes we identified. Despite the increased risk of unfavourable group dynamics (such as groupthink and group polarization) in difficult elicitation settings (such as two-staged problems of prioritizing risk then quantifying risk), our results suggest that these group dynamics did not dominate expert judgements. Structured, facilitated discussions may help avoid unfavourable outcomes.

### Reducing variability among experts

Some authors have proposed group processes as a mechanism to make expert responses more consistent, but such processes have sometimes been shown to yield consistency at the expense of accuracy via a phenomenon with the Orwellian title “groupthink” [22–24]. Groupthink often occurs when conformity within the group increases through conflict avoidance and suppression of dissent. Dominant personalities can also control the discussion and direct responses towards their point of view, introducing bias, as can expertise dominated by a particular field [6, 21]. However, groupthink (as well as group polarization) also leads to increased confidence in judgements because of corroboration from other group members [22]. We did not find evidence of groupthink in our results. We combatted this known problem in group elicitation by including pre-workshop individual responses as anchors, encouraging debate and defense of opinion among experts from diverse professions and fields, and forcing experts to provide post-workshop scores individually. These techniques have been shown to retain a healthy level of variability among experts after a group process [6, 14, 19]

The design of our workshop was specifically tailored to counteract groupthink. First, allowing experts to provide their estimates individually reduces the chance for pressure from dominant voices to sway their reporting [7]. Second, effective facilitators can control the time taken up by any individual voice and allow others to assess claims and judgements [1]. Third, giving experts an opportunity to listen to each other, assess and cross examine judgements in a structured facilitated setting reduces the effect of individual dominance and improves average performance [6, 13]. Exposing subject experts to diverse yet complementary expertise provides a more holistic consideration of the situation and reduces discipline dominance [21]. For example, we found that marine recreation experts reassessed their ranking to include climate change as a top five concern when exposed to fisheries, aquaculture, and biodiversity experts. Marine recreation in Tasman and Golden Bays is dependent on marine organisms, which was a specialty of the experts of the other three subjects.

The potential list of human impacts on coastal environments is immense and diverse, without an established nomenclature, and made up of generic activities, each of which includes many types of activities (such as agriculture, fisheries, etc.) and specific stressors (such as direct capture, ship strikes, sediment runoff, etc.). Experts tasked with identifying the more important risks must wade through this list, and individual experts may have different ways of describing and identifying risks even when describing the same phenomena [41]. To experts more accustomed to thinking about issues at landscape scales, broader activities may be more salient and immediately available for recall, whereas experts accustomed to investigating direct harm to wildlife may be more comfortable considering specific stressors. Navigating the resulting linguistic uncertainty among experts (where uncertainty is embedded in the terms used) can be mitigated through experts having the opportunity to clarify meaning and understanding [9]. We found that in multiple cases, before the workshop different experts identified multiple, varied activities as prominent risks because of their contribution to a particular stressor, whereas during the workshop with facilitated discussion experts were able to agree on consistent terminology pointing to the dominant stressor and converge on a shared model of how activities pose risk. The similarity of language used between experts is thought to be an important factor in leading to convergent mental models of a given problem (such as understanding the prominent risks to a coastal ecosystem), which can help move towards a comprehensive understanding of that problem [30, 42]. For example, individual experts pointed to agriculture, forestry, general land clearing (and other land uses), as well as dredging activities as important risks because of their role in sedimentation and estimated impact from these. Our facilitated workshop allowed experts to agree on consistent, specific terminology, highlighting the importance of sedimentation itself as a major risk across all ecosystem services.

Our finding of increased numerical consistency among experts suggests that the workshop also helped experts reassess their individual judgements about specific risks. More extreme views about the severity (or lack of severity) of impact from risks were moderated when confronted with challenges from other experts in our workshop, a finding found in other cases [13]. Though pressure from dominant voices might also have this effect, asking experts to provide their estimates individually likely mitigated this possibility [7].

Whether the findings of our case study that increased numerical consistency among experts after a workshop is generalizable to other workshop settings will likely depend on the skill of the facilitator, makeup of experts involved, including whether experts with pre-existing animosity are included, and whether contrasting advocates are involved in the workshop [7]. Any factor that increases the likelihood of pre-existing views becoming reinforced despite opposing evidence could counter factors encouraging convergence of judgements among experts, and contribute to polarization among group members [28]. The design of expert elicitations is important to reduce the probability that biases regulate expert responses [2, 9, 43]. Well-designed workshops thus are an important component of effective expert judgement elicitation [2, 6].

### Increasing subjective uncertainty within experts

One of the most prevalent problems with eliciting expert advice is contending with over-confidence [2, 19]. Experts are highly regarded individuals with high levels of training, qualifications, and/or experience. Society, and experts themselves, expect reliable performance as a result [6]. Status and expectations can lead to experts displaying excessive confidence in the accuracy of their beliefs [6]. In expert elicitations, overconfident experts provide estimate intervals that do not capture the true value they are estimating [19]. Given the prevalence of this form of overconfidence, methods to encourage experts to consider larger intervals for a given level of confidence are useful.

Some specific interventions applied to individual experts, such as asking experts to provide confidence intervals that meet a confidence cutoff, which we applied here, can force experts to reassess their intervals and provide larger intervals [19]. Similarly, exposing experts to each other in workshop settings as we did here is known to force a reevaluation of estimate intervals, increasing the estimate interval [6, 14]. This study utilizes interventions at the group level and, because estimates were collected before and after the workshop, provides evidence that group workshops can reduce overconfidence.

We used a before-after design to understand the influence of group elicitation on expert responses. Though this provides evidence of influence, a before-after-control-impact (BACI) design would provide stronger evidence [44]. By comparing against a control group, we would be able to rule out potential competing explanations for our results (that experts are more consistent but individually more unsure in their judgment after group deliberation), such as individual experts having more time to reflect on the questions. To truly understand if workshops can increase the consistency in experts’ identification of risks and decrease overconfidence in precision of risk, further cases documenting these effects are needed. However, this paper builds on the literature showcasing the role of workshops in generating good expert opinion.

## Conclusion

Expert elicitation is a valuable tool in situations where data is sparse and decisions urgent, and has the promise to be more valuable to tackle problems where a problem is not fully characterized beforehand. Where experts are needed both to provide context and estimates, evidence-based designs are needed to ensure that high quality expert judgements are captured. Through our case study, we show that in understanding cumulative impacts on ecosystem services, where prominent risks are not known beforehand, individual expert assessment followed by group deliberation can both increase consistency in experts’ identification of risks and decrease overconfidence in estimate intervals of impact. We argue that given their favorable effects on expert uncertainty, any study involving expert elicitation to deal with highly uncertain problems should strongly consider the use of expert workshops to improve expert risk estimates.
