# Correction: Group elicitations yield more consistent, yet more uncertain experts in understanding risks to ecosystem services in New Zealand bays

**DOI:** 10.1371/journal.pone.0190326

**Published:** 2017-12-20

**Authors:** Gerald G. Singh, Jim Sinner, Joanne Ellis, Milind Kandlikar, Benjamin S. Halpern, Terre Satterfield, Kai Chan

In the Author Contributions section, Jim Sinner (JS) should not be listed as one of the persons who contributed to funding acquisition.

There are errors in the Funding section. The correct funding information is as follows: The Ministry of Business, Innovation and Employment (grant MAUX1208) funded the elicitation process and workshop, but had no role (outside the authors) in study design, data analysis, decision to publish, or preparation of the manuscript.

There are errors in the Acknowledgements section. The correct Acknowledgements are as follows: We would like to thank Prof. Murray Patterson of Massey University for funding and the Cawthron Institute for their contribution of time and meeting space for this research. Special thanks to Mark Newton, Dana Clark, Sarah Klain, and Paige Olmsted for helping with the workshop. We would also like to thank the experts who took part in this study.
